# A Method for Predicting Alfalfa Biomass Based on Multimodal Data and Ensemble Learning Model

**DOI:** 10.3390/plants15050815

**Published:** 2026-03-06

**Authors:** Yuehua Zhang, Zhaoming Wang, Zhendong Tian, Haotian Deng, Jungang Gao, Chen Chen, Wei Zhao, Xiaoping Ma, Xueqin Ding, Haoran Yan, Liu Yang, Hui Xie, Qing Li, Fengling Shi

**Affiliations:** 1 College of Grassland Science, Inner Mongolia Agricultural University, Hohhot 010018, China; yuehua_zhang2023@emails.imau.edu.cn; 2 Technology R&D Center, M-Grass Ecology and Environment (Group) Co., Ltd., Hohhot 010010, China; wzhaoming0612@163.com (Z.W.); wstzd213@163.com (Z.T.); occo4565@163.com (C.C.); 13474700474@163.com (W.Z.); d9701056275@163.com (X.D.); yanhaoran1018@163.com (H.Y.); mnxiehui@126.com (H.X.); 3 Key Laboratory of Cultivated Land Capability Conservation and Improvement in Agro-Pastoral Ecotone Ministry of Agriculture and Rural Affairs of the People’s Republic of China, Hohhot 010010, China; 4 College of Agriculture, Nanjing Agricultural University, Nanjing 210095, China; 2025222001@stu.njau.edu.cn (H.D.); qingli@njau.edu.cn (Q.L.); 5 Inner Mongolia Grass Digital Agricultural Ecology Co., Ltd., Hohhot 010010, China; allfly@126.com (J.G.); xiaocaoshuzi@163.com (X.M.); ylwilll@126.com (L.Y.)

**Keywords:** alfalfa, multispectral, LiDAR, machine learning, biomass prediction

## Abstract

Accurate alfalfa biomass prediction is crucial for pasture management and sustainable livestock production. However, traditional methods often perform poorly under complex field conditions. To address the limited prediction accuracy of traditional methods under complex planting environments, this study proposes an alfalfa biomass prediction method combining multispectral and LiDAR data with ensemble learning model. Based on the multispectral images acquired by unmanned aerial vehicle (UAV) and airborne LiDAR data, the spectral features, three-dimensional structural features, and their interaction features are systematically extracted at the quadrat scale, and a high-quality modeling dataset is constructed by feature selection. Secondly, an ensemble model for alfalfa biomass prediction was constructed, which was composed of random forest, extra trees, and histogram gradient boosting. After model training, the coefficient of determination (R^2^) of the integrated model on the test set reached 0.813, and the root mean square error (RMSE) and mean absolute error (MAE) were 0.178 kg m^−2^ and 0.146 kg m^−2^, which were significantly better than those of similar single models. Under feature combinations, the fusion model was better than that of spectral indices only (R^2^ = 0.773) and LiDAR traits only (R^2^ = 0.576), and the model achieved the highest accuracy from bud emergence to early flowering (R^2^ = 0.917). The overall prediction error of the model was approximately normal distribution, and the absolute error of more than 65% of the samples was less than 0.2. However, there was still a trend of underestimation in the high biomass interval. This research showed that the multimodal data fusion and ensemble learning method could achieve high-precision prediction of alfalfa biomass, which provided reliable technical support for pasture resources monitoring and precision agriculture management.

## 1. Introduction

As a legume perennial grass, alfalfa has become an indispensable high-quality forage resource in the global agricultural and animal husbandry production system due to its high protein content, strong nitrogen fixation efficiency, and wide ecological adaptability [1,2]. Accurate prediction of alfalfa biomass is the core technical link for implementing precise management, optimizing forage harvest regulation, and quantifying ecological and economic value [3], and its prediction accuracy is directly related to the sustainable production efficiency of alfalfa ecosystem and the level of intensive development of animal husbandry [4]. In northern China, particularly in Inner Mongolia, alfalfa is a key forage crop supporting grassland-based livestock production systems. The region is characterized by extensive pastoral and agro-pastoral systems, where high-quality forage supply is essential for maintaining livestock productivity and ecological sustainability [5]. However, due to the coupling effect of multiple factors such as genotype, soil physical and chemical properties, climatic factors, and field management measures, alfalfa biomass shows significant spatial and temporal heterogeneity and nonlinear characteristics [6,7], and traditional prediction methods are difficult to meet the high-precision requirements in complex scenarios. Therefore, the construction of an efficient and stable biomass prediction model has become a research hotspot and key issue in alfalfa production.

The prediction methods of alfalfa biomass are mainly divided into three categories: traditional experimental measurement methods, statistical modeling methods, and single machine learning modeling methods [8]. Although traditional experimental measurement methods (such as the whole-plant harvesting method and drying and weighing method) can provide direct measured biomass data, the inherent defects of destructive sampling and its time-consuming nature make it impossible to achieve large-scale and dynamic continuous monitoring, and the systematic error and random error in the sampling process can easily significantly affect the accuracy of the results [9]. Statistical modeling methods (such as multiple linear regression and principal component regression) realize prediction by constructing a linear correlation model between biomass and environmental factors. Although they have the advantage of simple calculation, it is difficult for them to describe the complex interaction between multiple factors, and the prediction accuracy decreases significantly under non-stationary environmental conditions [10]. In recent years, machine learning models have been widely used in the field of biomass prediction due to their powerful nonlinear fitting and feature learning capabilities [11]. Among them, support vector machine [12,13], random forest [14,15], artificial neural network [16,17], and other models have made some progress in biomass prediction. Based on multi-source remote sensing and ground survey data, Bui et al. [18] constructed an intelligent optimized hybrid machine learning model for forest aboveground biomass estimation, and achieved the best performance in the case of northern Vietnam, which was significantly better than traditional methods. Hu et al. [19] used UAV hyperspectral imagery and machine learning modeling, and the R^2^ of the random forest method reached 0.95, which achieved the fast and high-precision estimation of milk vetch biomass. Tunca et al. [20] constructed a UAV-based machine learning framework that integrates multispectral and canopy structural information to achieve accurate, non-destructive estimation of sorghum above-ground biomass across different irrigation regimes. KC et al. [21] extracted 13 vegetation indices from multispectral images acquired by UAS, selected the optimal six indices based on VIF feature selection, and then used XGB to construct regression models to estimate rye biomass. After fusing field structure features, the coefficient of determination was increased to 0.82, achieving high-precision and spatialized biomass prediction. Swain et al. [22] used PlanetScope high-resolution images combined with 10 vegetation indices, three soil nutrient factors extracted by PCA, and a total of approximately 36 environmental and crop parameters to construct a total of more than ten machine learning models to predict the total biomass and yield of rice, among which Cubist’s R^2^ reached 0.88. However, there are still limitations that cannot be ignored in a single machine learning model: the SVM model is prone to overfitting in high-dimensional data space [23], the RF model is not robust to noise data [24], and the ANN model has a high demand for training sample size and slow convergence speed [25]. At the same time, the existing research is mostly based on spectral data sources to construct prediction models, ignoring the complementarity and synergy of information contained in different data sources in the spatial range, which leads to an incomplete analysis and description of the biomass formation mechanism, and makes it difficult to adapt to the dynamic prediction needs in complex field environments.

The development of multimodal data fusion technology and machine learning methods provides a new technical path to solve the above problems. With the advantages of active detection and penetration, LiDAR technology can penetrate the closed crop canopy, obtain high-precision 3D point cloud data, and directly quantify key geometric parameters such as vertical stratification density [26]. Multispectral imagery can provide rich spectral reflectance and texture features, which can invert crop physiological and biochemical indicators, and effectively represent the enrichment information of biomass [27]. Chen et al. [28] proposed a comprehensive index VHI combining multispectral and LiDAR data, which achieved a low root mean square error (RMSE) of 272 g/m^2^ in the estimation of alfalfa biomass in mountainous areas, which was significantly better than the accuracy of the single LiDAR height index (322 g/m^2^). Wu et al. [29] used LiDAR and multispectral data to invert cotton plant height and chlorophyll content, and constructed an XGBoost integrated model based on multiple time series growth characteristics to predict cotton yield R^2^ up to 0.802, which was significantly better than the model containing only a single time series feature. Chen et al. [30] confirmed through experiments that the fusion of UAV multispectral and LiDAR data could effectively alleviate the spectral saturation effect in the later stage of corn growth, and the integrated model based on random forest reached R^2^ 0.86 in the estimation of nitrogen use efficiency, which was 20.21% higher than the average estimation accuracy of a single data source. Therefore, the construction of multimodal data based on multispectral and LiDAR can complementarily characterize the key information of crop growth status and biomass formation process from different dimensions and scales, and provide solid data support for improving prediction accuracy. However, the application of multimodal data and machine learning models in the prediction of forage biomass such as alfalfa is still in its initial stage, and there is still a lack of systematic research.

Therefore, this study proposes a novel alfalfa biomass prediction framework integrating UAV multispectral imagery and airborne LiDAR data with an ensemble learning strategy. Unlike previous studies that mainly rely on single remote sensing data sources or individual machine learning models, the proposed methodology aims to establish a more systematic and adaptive modeling framework by integrating multimodal feature interaction and heterogeneous ensemble learning under multi-stage field conditions. The main contributions of this study are summarized as follows:

(1) A multimodal feature system integrating UAV multispectral imagery and airborne LiDAR point cloud data was constructed, systematically characterizing alfalfa canopy from both spectral and three-dimensional structural dimensions.
(2) A heterogeneous ensemble learning framework combining Random Forest, Extra Trees, and Histogram Gradient Boosting was developed to enhance prediction accuracy and model robustness under limited sample conditions.
(3) The effects of different feature combinations (spectral-only, LiDAR-only, and fused features) on biomass prediction performance were quantitatively evaluated.
(4) The temporal variation of model accuracy and feature importance across different growth stages was systematically analyzed, revealing dynamic changes in spectral and structural contributions.
(5) A comprehensive evaluation including regression analysis, residual distribution analysis, and robustness assessment was conducted to validate the reliability and stability of the proposed model.

Using the above method, this study verified the advantages of the proposed method in terms of accuracy, robustness, and applicability, which provided reliable technical support for accurate monitoring and intelligent management of alfalfa biomass.

## 2. Materials and Methods

### 2.1. Experimental Site

**Figure 1:**
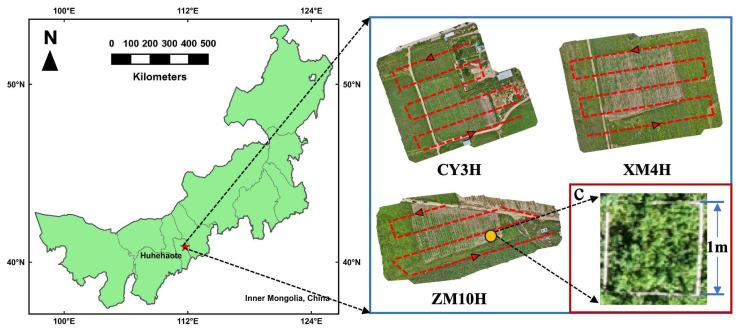
Location of the experimental sites in Hohhot, Inner Mongolia, China. Field experiments were conducted at two sites (Mongolian Grass Seed Industry Center Base and Heimawa Base) with identical experimental designs. The red dashed line represents the flight path.

**Figure A1:**
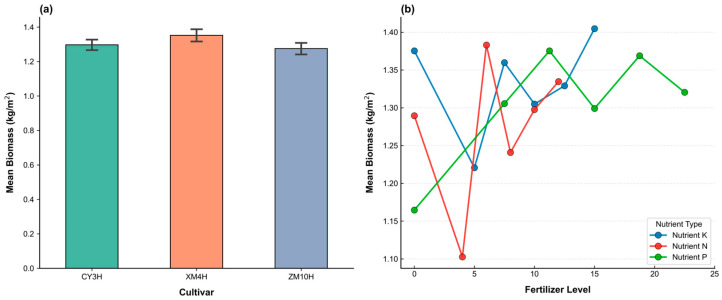
Average biomass across different cultivars and fertilizer levels. (**a**) Mean biomass for three cultivars (CY3H, XM4H, ZM10H) averaged across all fertilizer levels. (**b**) Mean biomass for different fertilizer levels, averaged across the three cultivars.

The experiment was conducted at the Mongolian Grass Seed Industry Center Base (40.90° N, 111.78° E) and the Heimawa Base (40.39° N, 111.54° E) in Hohhot, Inner Mongolia, China (Figure 1). Both sites followed identical experimental designs, including the same alfalfa varieties, fertilizer treatments, and management practices. The total area of the experimental field was 30 mu (10 mu for each variety). The experimental alfalfa varieties were Grassland No. 3 mixed flower alfalfa (CY3H), Zhongmu No. 10 alfalfa (ZM10H) and Xinmu No. 4 alfalfa (XM4H), and the planting density was 1 kg/mu (1 mu = 0.0667 ha). In this study, a one-factor-at-a-time experimental design was adopted to investigate the effects of fertilizer application on alfalfa biomass. Specifically, for each treatment, only one fertilizer (N, P, or K) was varied across different levels, while the other two fertilizers were maintained at their conventional application rates. Nitrogen (N), phosphorus (P), and potassium (K) fertilizers were applied at six gradient levels based on the conventional application rate (0%, 50%, 75%, 100%, 125%, and 150%). For nitrogen treatments, N was applied at 0, 4, 6, 8 (conventional rate), 10, and 12 kg·mu^−1^, while P and K were kept at their conventional application rates. For phosphorus treatments, P was applied at 0, 7.5, 11.25, 15 (conventional rate), 18.75, and 22.5 kg·mu^−1^, while N and K were maintained at their conventional levels. For potassium treatments, K was applied at 0, 5, 7.5, 10 (conventional rate), 12.5, and 15 kg·mu^−1^, while N and P were kept at their conventional application rates. Each fertilization treatment was arranged with three replicates in a randomized block design, and each plot had an area of 64 m^2^ (8 m × 8 m). All plots were managed uniformly according to the local high-yield cultivation practices, except for differences in fertilizer application. During sampling, a quadrat (1 m × 1 m) was taken from each plot. Biomass yield was measured for each plot based on quadrat sampling, and the effects of cultivar, fertilizer type, and fertilizer level on biomass production were further analyzed (Figure A1).

### 2.2. Data Acquisition

**Figure 2:**
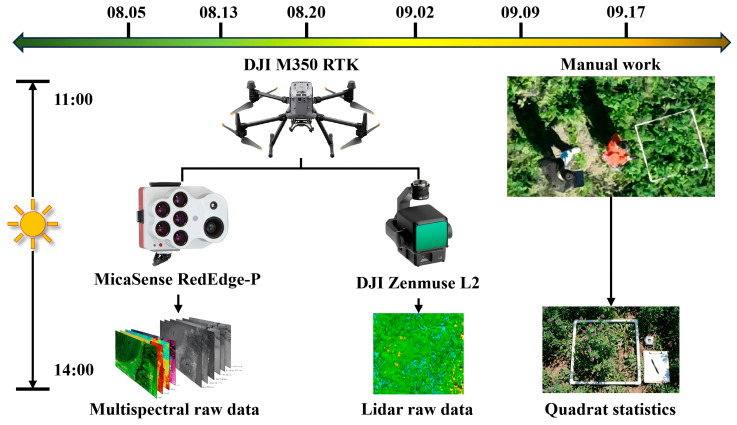
Data acquisition process.

Data collection was carried out from August to September 2025 (Figure 2). In this study, multiple aerial surveys were carried out during the alfalfa growing season in three plots of CY3H, XM4H, and ZM10H, and a total of six simultaneous field surveys were carried out (5 August, 13 August, 20 August, 2 September, 9 September, and 17 September, respectively). The UAV was equipped with a multispectral camera and an airborne laser radar. The data collection was arranged between 11:00 a.m. and 2:00 p.m., when the weather was clear and the ground wind speed was less than 5 m/s.

LiDAR point cloud data were collected by the Chansi L2 LiDAR mapping system of Shenzhen DJI Innovation Technology Co., Ltd. (Shenzhen, China). The multispectral data were collected by RedEdge-P multispectral sensor Suite system (Table 1) from MicaSense, (Seattle, WA, USA). In each acquisition period, multispectral images and airborne LiDAR data were acquired, and RTK real-time differential positioning was enabled to achieve centimeter-level registration without ground control points.

After the collection was completed, the alfalfa field biomass was measured. In each treatment area, three duplicate quadrats (each with an area of 1 m × 1 m) were randomly set up according to the diagonal method, and the quadrats were placed after avoiding the edge of the plot for at least 1 m. The shoot plants were cut evenly along the soil surface in each quadrat. All plant samples were collected and placed into paper bags and marked. The samples were taken back to the laboratory immediately. The attached soil and debris were removed through a quick rinse with tap water, and the surface water was gently blotted dry with absorbent paper. Then, the samples were dried in a 65 °C blast drying oven to constant weight (the difference between two consecutive weighing less than 0.01 g was considered as constant weight), and the dry weight was accurately weighed using an electronic balance. The alfalfa biomass of each quadrat was converted to biomass per unit area (g·m^−2^) by dry weight, and the measured values of three quadrats were used as triplicates of the treatment for subsequent statistical analysis.

### 2.3. Data Processing

#### 2.3.1. Data Splicing and Synthesis

**Figure 3:**
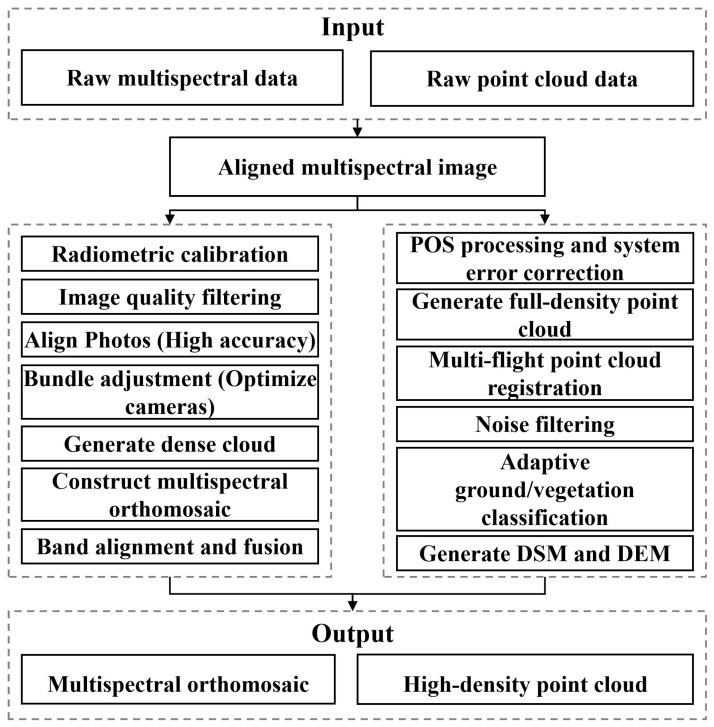
Data stitching and synthesis.

In order to ensure the geometric and radiometric consistency of multi-source data, Agisoft Metashape and DJI Terra software (Version 5.1) [31] were used to complete the mosaic, alignment, and fusion of multispectral images and LiDAR point clouds, respectively. All the processing processes were performed under a unified coordinate reference (Figure 3).

The acquired alfalfa multispectral images were processed and stitched using Agisoft Metashape (Figure 3). Firstly, the original images of each band (blue, green, red, red edge, and near infrared) were imported into the software, and the data of participating illumination sensors in the camera were automatically read. Then, the radiometric correction was performed, including calculating the reflectance coefficients of each band based on the reflectance calibration board, compensating for the illumination changes during flight by using the downlink illumination sensor (DLS) data, and combining the dark current and vignetting correction of the lens to obtain a physically consistent reflectance image. After radiometric correction, the quality of the image was screened (image quality > 0.5) to eliminate blurred or abnormal exposure data. Then, the accuracy was set to high in the Align Photos module, and the sparse point cloud was constructed and the relative pose of the image obtained. Check points were combined for external orientation constraints, and the bundle adjustment optimization was completed by Optimize Cameras. After the camera orientation was completed, a dense point cloud was generated for each band, the filtering level was set to medium, and the multispectral orthophoto was reconstructed. Finally, Raster Calculation was used to complete the registration and fusion of each band, and the radiometric corrected multispectral orthophoto with high spatial resolution was output, which was used for the subsequent calculation of alfalfa vegetation index and growth characteristics analysis.

The acquired alfalfa LiDAR data were processed by DJI Terra. Firstly, the original airborne LiDAR data (including IMU and GNSS trajectories) were imported into the software, and the system performed a preliminary track calculation according to the POS data to obtain the initial point cloud of time synchronization. Then, systematic error correction, including boresight offset correction and roll, pitch, and yaw angle compensation, was performed to improve the geometric consistency of the point cloud. In the point cloud generation stage, the LAS quality was set to 100% to ensure that the output point cloud retained all the effective laser echo information and the highest density of spatial sampling, which was used to enhance the three-dimensional detail representation of the alfalfa canopy structure. The point cloud reconstruction module was used to complete the stitching and registration of multiple flight datasets. The overlapping area was iteratively matched by using trajectory constraints and multi-view geometric features, and the check points were incorporated into the overall optimization to further improve the absolute spatial accuracy of the point cloud. After mosaicking, noise filtering and classification were performed. Statistical filtering and height thresholding were applied to remove isolated noise and outliers. Ground points were classified and a Digital Elevation Model (DEM) and Digital Surface Model (DSM) were generated accordingly. Vegetation point clouds were extracted based on the classification results. Canopy height metrics were derived from Height Above Ground (HAG) after normalization, and all structural features were calculated based on relative height within each quadrat. The processed LiDAR data with unified coordinate system and complete density were used for the subsequent canopy structure analysis and biomass-related feature extraction. The stitched results (Table 2) were obtained by stitching and synthesizing all the collected multispectral images and LiDAR point cloud data.

After data stitching and integration, two types of final data were formed in this study: multispectral image data and LiDAR point cloud data (Table 2). The synthesized multispectral image data were used for subsequent spectral reflectance analysis and vegetation index construction. Correspondingly, the LiDAR point cloud with reflectance and other indicators can support the point density statistics and the extraction of vegetation height characteristics. The final synthesized data set has high quality in terms of spectral information and spatial structure information, which provides sufficient data basis for subsequent model feature construction and analysis.

#### 2.3.2. Data Registration

**Figure 4:**
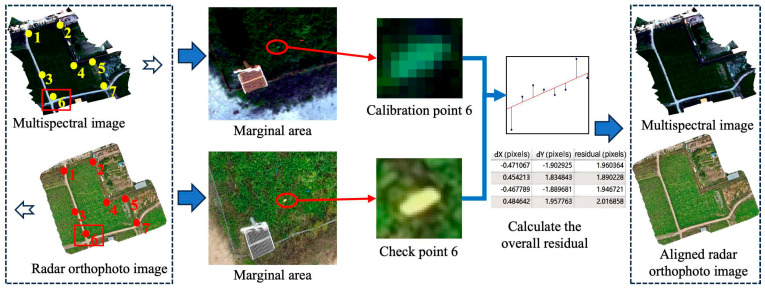
Data registration process. The black and white dovetail arrow indicates the offset of the two kinds of data in spatial position. Yellow and red points are marked check points.

In order to ensure the spatial consistency of multi-source remote sensing data, multispectral images were used as the reference to calibrate the position of the LiDAR orthophoto, so as to produce the unified coordinate framework of point clouds and multispectral images. Taking CY3H as an example (Figure 4), firstly, the Georeferencer tool was invoked in the QGIS environment to set the LiDAR orthophoto as the target image and the multispectral orthophoto as the reference image. Seven check points (calibration points 1–7) with uniform distribution, stable geometric structure, and easy identification were selected from the two types of images, including the four boundaries of the region and the central part of the region. All the check points were visually inspected to ensure accurate correspondence. A polynomial transformation model was applied to geometrically align the registered images. Specifically, a set of corresponding control points between the multispectral image and the LiDAR data was used to establish the transformation relationship. The polynomial coefficients were estimated using a least-squares fitting approach to minimize the geometric error between the two datasets. Depending on the degree of geometric distortion and the spatial scale of the study area, either a second-order or third-order polynomial model was adopted. The model order was selected by evaluating the residual error of the control points, aiming to achieve a balance between fitting accuracy and overfitting risk. Lower-order models were preferred when the distortion was limited, while higher-order models were used when more complex spatial distortions were observed. The root mean square error (RMSE) was calculated automatically by the tool to evaluate the registration accuracy. By gradually adding, deleting, or fine-tuning the check points, the transformation model was iteratively optimized until the RMSE was lower than the preset threshold to ensure that the spatial consistency between images met the accuracy requirements of agricultural remote sensing analysis.

After image-level registration, the spatial reference system of the LiDAR orthophoto was highly consistent with that of the multispectral imagery. The geometric transformation parameters obtained during the registration process were subsequently applied to the original LiDAR point cloud data, enabling spatial alignment between the point cloud and the multispectral image. Both datasets were unified under the same geographic coordinate framework, allowing seamless matching at the quadrat scale. This provides a reliable basis for the joint analysis and multimodal fusion modeling of alfalfa structural features (e.g., height, stratification proportion, coverage) and spectral features (e.g., reflectance and vegetation indices).

To further ensure multimodal consistency, multispectral imagery and LiDAR point clouds were acquired during the same UAV flight campaigns, minimizing temporal discrepancies between spectral and structural measurements. Both datasets were georeferenced using RTK positioning under a unified coordinate reference system. The polynomial-based geometric transformation was iteratively optimized until the spatial registration error (RMSE) was controlled within centimeter-level accuracy. Therefore, any residual temporal or spatial misalignment is unlikely to significantly influence biomass modeling at the quadrat scale.

#### 2.3.3. ROI Labeling and Data Fusion

**Figure 5:**
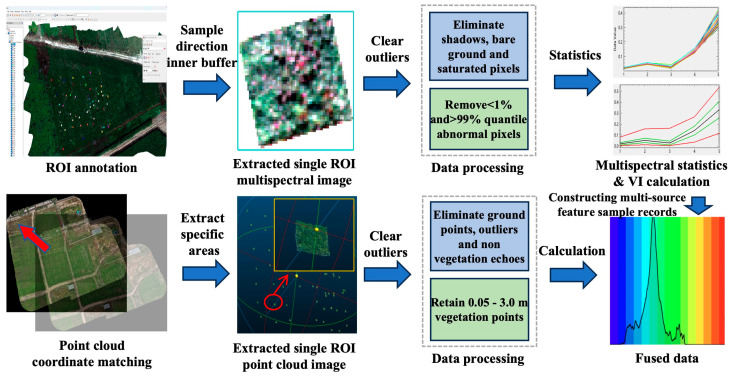
ROI labeling and data fusion process. The red arrow represents the true position after correcting the coordinate offset.

After multi-source data registration, the quadrat was labeled using ENVI 5.6 software. The location information of 54 ROI regions was labeled for each period of images, and a total of 162 ROI were obtained for each period. A sample data set corresponding to the ground measured biomass was constructed under a unified spatial reference framework to realize the accurate correlation between UAV multimodal features and ground truth values, and the multispectral and LiDAR data fusion data were constructed (Figure 5).

After the ROI labeling was completed, the multispectral image and point cloud data of the corresponding ROI region were extracted and processed, respectively (Figure 5). Considering that the UAV image and point cloud may have geometric distortion and pixel mixing at the edge of the field of view, in order to reduce the influence of boundary effect on the feature statistics, an inward buffer of 5–10 cm was applied to each quadrat as the spatial unit for subsequent feature extraction. Within the ROI, the quality of the LiDAR point cloud and the multispectral image were filtered and masked, respectively. In the LiDAR part, ground points, obvious outliers, and non-vegetation echoes were eliminated using a rule-based filtering strategy. Specifically, ground points were separated using a progressive morphological filtering approach [32], and outliers were removed using neighborhood-based statistical filtering [33]. In addition, a height threshold (0.05–3.0 m) was applied to retain vegetation points. In the multispectral part, abnormal pixels affected by strong shadow, bare ground, or sensor saturation were eliminated using a combination of threshold-based masking and quantile filtering [34]. Specifically, non-vegetation pixels were excluded using a vegetation index threshold (NDVI < 0.20), which has been widely used for vegetation masking in UAV-based multispectral studies. Shadowed pixels were removed based on low near-infrared reflectance (NIR < 0.05), as NIR bands are highly sensitive to vegetation structure and illumination conditions. Saturated pixels were identified and excluded when reflectance values in any band exceeded 0.95. Furthermore, quantile filtering was performed within each ROI for each band, and pixels with reflectance values below the 1st percentile or above the 99th percentile were excluded to reduce the influence of extreme values. Similar percentile-based filtering strategies have been applied in multispectral data analysis to suppress noise and improve the robustness of feature extraction [35]. This procedure ensured that the spectral features were mainly derived from homogeneous vegetation cover areas. On this basis, each quadrat was used as a spatial unit to construct sample records containing multi-source information. Each sample record contains a unique quadrat ID, structural features extracted from LiDAR point clouds, multispectral band statistics and vegetation indices, and interactive features formed by the combination of spectral and structural information. Environmental and management metadata, such as sampling date, fertilizer treatment type, and flight batch are also included in the sample record. Finally, 270 valid samples were taken as the data set, and the sample naming follows the uniform rules (Table 3).

### 2.4. Feature Extraction

In order to comprehensively describe the three-dimensional structural characteristics, spectral response characteristics, and spatial heterogeneity of alfalfa canopy, we systematically extracted multi-dimensional features from LiDAR point clouds and multispectral images at quadrat scale, and constructed a feature system based on spectral and structural fusion. All features were calculated within the labeled ROI to ensure that their spatial ranges strictly corresponded to the measured biomass.

In the multispectral part, the Blue, Green, Red, RedEdge, and NIR bands were used as the basis to extract spectral features reflecting the spectral response and physicochemical properties of the canopy. Firstly, the mean, median, standard deviation, minimum, and maximum values of each band were calculated in the ROI to describe the overall level, dispersion degree, and extreme performance of spectral reflectance. At the same time, the 10th to 90th percentile reflectance was extracted based on quantile statistics to fully reflect the internal structure of the pixel value distribution, so as to more fully reflect the reflectance variation within the canopy. In order to characterize the spatial heterogeneity of the canopy and the difference of vegetation cover, the histogram of pixel values in each band was constructed, and the characteristics related to the distribution shape, such as the location and frequency of the main peak, and the distribution width, were extracted to describe the changes of spectral response under different vegetation density and canopy structure. On this basis, a series of commonly used vegetation indices, including NDVI, NDRE, GNDVI, MSR, EVI, and SAVI, were further calculated (Table 4). For each ROI, the mean value of each vegetation index was computed based on the valid pixels after masking, and used as input features for subsequent regression analysis. These indices are closely related to chlorophyll content, photosynthetic activity, and canopy structure density, and are important spectral indices for retrieving aboveground biomass. In addition, geometric features such as the sample area, the number of effective pixels, and the total number of pixels were extracted.

In LiDAR data processing, canopy structure parameters were extracted based on the height aboveground (HAG) information after normalization. Firstly, a series of height statistics were calculated to describe the overall height level and distribution characteristics of alfalfa canopy, including the maximum height Hmax, the average height Hmean, the standard deviation Hstd, and the coefficient of variation Hcv. Furthermore, several percentile heights including Hq05, Hq10, Hq25, Hq50, Hq75, Hq90, and Hq95 were extracted to describe the shape and tail characteristics of canopy height distribution. In addition, the height interquartile range HIQR was used to quantitatively describe the degree of height dispersion, which reflected the puffiness and layer-sense of canopy from the side. On the basis of height characteristics, point density and coverage related indicators were further calculated to characterize the density and canopy closure of vegetation growth. Specifically, it included the total point density and vegetation point density in the ROI, as well as the point cloud coverage proportion at different height thresholds (e.g., H > 0.3 m and H > 1.0 m), thereby reflecting the degree of canopy closure at different altitude levels. Considering the obvious vertical stratification characteristics of alfalfa canopy, the HAG range was divided into several height intervals (such as 0–0.5 m, 0.5–1.0 m, 1.0–1.5 m, etc.), and the proportion of point cloud number in the total vegetation points in each altitude level was calculated to quantify the vertical structure distribution of the canopy. In addition, the mean value, standard deviation, and histogram features of laser echo intensity were extracted to describe the complexity of canopy structure, vegetation density, and laser penetration ability. Together with height and density features, a complete index system for describing the three-dimensional structure of alfalfa was formed. However, aboveground biomass is influenced not only by structural traits but also by physiological variability that is more directly captured by spectral responses [36]. In addition, under low-biomass conditions, LiDAR returns may be affected by ground interference and limited vertical differentiation, which can reduce predictive sensitivity [37]. Therefore, LiDAR-based features used alone may show relatively lower performance compared with multispectral or multimodal approaches.

In order to enhance the model’s ability to express the structure-spectral coupling relationship, this study constructs a variety of interactive features based on the above single-source features, and combines the LiDAR structural parameters with the multispectral spectral parameters. At the same time, the interaction between height percentile and spectral statistics is considered, and the joint feature of vertical stratification proportion and vegetation index is introduced to describe the comprehensive influence of vegetation at different elevation levels on spectral absorption and reflection. In order to reduce redundant information, improve model training efficiency, and reduce the risk of overfitting, we selected features based on the built-in feature importance index of the Random Forest model. In the random forest regression model, feature importance was quantified based on the reduction of node impurity measured by mean squared error (MSE). For a given node *t*, impurity was defined as the variance of the target variable within the node:

(1) $$I(t)=\frac{1}{N_{t}}\sum_{i\in t}{\left(y_{i}-{\bar{y}}_{t}\right)}^{2}$$

where $N_{t}$ is the number of samples in node *t*, $y_{i}$ denotes the observed aboveground biomass, and ${\bar{y}}_{t}$ is the mean value of the samples in node *t*.

When node t is split into left and right child nodes $t_{L}$ and $t_{R}$ using feature j, the impurity reduction contributed by this split is calculated as:

(2) $$\Delta I(t,j)=I(t)-\frac{N_{t_{L}}}{N_{t}}I(t_{L})-\frac{N_{t_{R}}}{N_{t}}I(t_{R})$$

The importance of feature j for the *m*-th decision tree is obtained by summing the impurity reductions over all nodes where feature j is used for splitting:

(3) $$FI_{j}^{\left(m\right)}=\sum_{t\in T_{m}:v(t)=j}\Delta I(t,j)$$

Finally, the overall importance of feature j in the random forest model is computed as the average importance across all trees:

(4) $$FI_{j}=\frac{1}{M}\sum_{m=1}^{M}FI_{j}^{\left(m\right)}$$

This impurity-based feature importance reflects the contribution of each feature to reducing prediction error in the regression task. In this study, feature importance derived from Random Forest (based on the reduction of mean squared error at each split) was adopted for feature screening. This tree-based importance measure does not require assumptions of linearity or specific feature distributions and is suitable for capturing nonlinear relationships and potential interactions among multimodal features [38,39]. In addition, Random Forest-based feature ranking has been widely applied in high-dimensional regression tasks due to its robustness and computational efficiency [40]. Since the final modeling framework in this study also includes tree-based ensemble methods, adopting Random Forest importance ensures methodological consistency between feature selection and model construction. Therefore, it provides a consistent and practical criterion for selecting informative variables in the present study. According to the calculated cumulative contribution rate of feature importance, the feature subset with a contribution rate of 95% was selected as the final input variable, and 49 features were finally selected as modeling features (Table 5) to balance the model complexity and prediction accuracy. The complete list of all features used in this study is provided in Table A5. All constructed features, including interaction features, were ranked based on Random Forest importance. Features with relatively low contribution, particularly most interaction features, were ranked lower and therefore contributed less to the final model. Many LiDAR-derived features were excluded during feature selection due to high collinearity or marginal contribution, and only the most informative structural descriptors were retained. The feature ranking and screening procedure is primarily used to reduce redundancy and to retain informative representative variables from correlated feature groups.

### 2.5. Machine Learning Model Construction

Based on the above feature parameters, the dataset was randomly divided into training set (229 samples) and test set (41 samples) according to the ratio of 85%:15%. The dataset was not partitioned according to growth stages. All samples from different phenological stages were combined into a unified dataset for model training, data splitting, and hyperparameter optimization. Therefore, the model was trained on mixed-stage data to learn generalizable relationships across growth stages. To evaluate the potential impact of random train-test splitting on model performance, repeated random splits with different random seeds were conducted (Table A1). The performance metrics exhibited only minor fluctuations across different splits, indicating that the proposed ensemble model is relatively stable and not sensitive to a specific data partition. In order to improve the accuracy and stability of biomass estimation driven by multimodal features, this study constructed a Biomass Ensemble Model based on heterogeneous tree model. The framework combines Random Forest (RF), Extra Trees (ET), and Histogram Gradient Boosting (HGB) algorithms to make full use of the complementary advantages of each algorithm in bias-variance characteristics. RF constructs multiple base learners by bootstrap sampling and feature subspace randomization, and its model structure has low variance and good noise immunity. On this basis, ET further enhances the randomness of node division, which improves the diversity of the ensemble model and helps to reduce the upper bound of the generalization error. HGB adopts a gradient boosting strategy based on feature binning, so that it can maintain high efficiency and prediction performance when dealing with high-dimensional nonlinear relationships and data with missing values. In this study, the weighted average soft voting strategy is used to achieve multi-model fusion. Note that the predicted values of the three base learners are ${\hat{y}}_{RF}$, ${\hat{y}}_{ET}$ and ${\hat{y}}_{HGB}$, respectively; the final prediction of the integrated model can therefore be expressed as:

(5) $$\hat{y}=w_{RF}{\hat{y}}_{RF}+w_{ET}{\hat{y}}_{ET}+w_{HGB}{\hat{y}}_{HGB}$$

The weights were determined based on performance evaluation within the training data, aiming to achieve a balance between bias and variance. The test set was used solely for final evaluation. To avoid numerical instability introduced by features of different scales during model training, RobustScaler [41] was applied to normalize all input features before feeding them into each sub-model.

Hyperparameter optimization was conducted using grid search combined with 10-fold cross-validation. The objective was to maximize the cross-validated coefficient of determination (R^2^). For the Random Forest and Extra Trees models, the tuned hyperparameters included the number of estimators (100–1500), maximum tree depth (None or 5–30), minimum samples required for node splitting (2–10), and minimum samples per leaf (1–10). For the Histogram Gradient Boosting model, the optimized parameters included learning rate (0.01–0.2), maximum tree depth (3–15), number of boosting iterations (100–1500), minimum samples per leaf (1–50), and L2 regularization strength (0–5). All models were optimized under identical cross-validation settings to ensure a fair and consistent comparison.

All the model training work was completed on the Ubuntu 20.04 system. The hardware environment includes an RTX 3090 graphics card (24 GB VRAM), an AMD Ryzen 9700X processor with 64 GB RAM, and the software environment includes Python 3.10, scikit-learn 1.5, and PyTorch 2.2. The coefficient of determination (R^2^), root mean square error (RMSE), and mean absolute error (MAE) were used as evaluation indicators:

(6) $$R^{2}=1-\frac{\sum_{i=1}^{n}{\left(y_{i}-{\hat{y}}_{i}\right)}^{2}}{\sum_{i=1}^{n}{\left(y_{i}-\bar{y}\right)}^{2}}$$

(7) $$\mathrm{RMSE}=\sqrt{\frac{1}{n}\sum_{i=1}^{n}{\left(y_{i}-{\hat{y}}_{i}\right)}^{2}}$$

(8) $$\mathrm{MAE}=\frac{1}{n}\sum_{i=1}^{n}|y_{i}-{\hat{y}}_{i}|$$

By comparing the values of R^2^, RMSE and MAE of different models, the most suitable model for predicting alfalfa biomass was selected.

## 3. Results

### 3.1. Model Parameter Optimization and Performance Comparison

In order to ensure the high stability and generalization ability of the alfalfa biomass prediction model, this study systematically optimized the parameters of the weighted ensemble model (Table 6), calculated the performance indicators of the model on the test set, and evaluated the model.

For the optimal hyperparameter configurations of random forest, extra trees, and HGB models (Table 6), each model employs a large number of base learners to improve prediction stability and reduce random error. By limiting the tree depth and setting the minimum split and the number of leaf node samples, the random forest achieves a balance between the model expression ability and the control of overfitting. The extra trees model does not limit the tree depth, and combines a stronger random splitting mechanism to enhance the diversity and generalization ability of the model. The HGB model adopts a smaller learning rate and a deeper iteration number, and introduces regularization constraints to achieve a stable stepwise optimization process. The above parameter configurations reflect the trade-off between complexity control and prediction performance of different ensemble learning strategies, and provide a robust parameter basis for model training. In order to compare the performance of the models more intuitively, the data set is modeled and the results are analyzed by using similar models (Table 7). For all machine learning models, hyperparameter tuning was performed using grid search combined with cross-validation on the training set. The reported results correspond to the best-performing configurations identified within predefined parameter ranges for each model. The artificial neural network (ANN) used in this study is a shallow feedforward neural network consisting of one hidden layer with 64 neurons. The hidden layer uses the ReLU activation function, and the output layer is a linear neuron for regression. The deep neural network (DNN) is implemented as a multilayer perceptron with three hidden layers containing 128, 64, and 32 neurons, respectively. Each hidden layer is followed by a ReLU activation function and dropout regularization (dropout rate = 0.2) to reduce overfitting. Both ANN and DNN models are trained using the Adam optimizer with a learning rate of 0.001 and mean squared error (MSE) as the loss function. Early stopping is applied based on validation loss to prevent overfitting.

Significant variations in prediction accuracy and error control are observed across different models (Table 7), with model performance demonstrating a progressive improvement from shallow neural networks to deep learning, traditional machine learning, and finally to ensemble learning. The shallow artificial neural network (ANN) exhibited the lowest predictive performance, yielding an R^2^ of 0.413, along with the highest RMSE (0.318 kg m^−2^) and MAE (0.234 kg m^−2^), indicating its limited capacity to capture the complex relationships between remote sensing features and biomass. By comparison, the deep neural network (DNN) achieved an R^2^ of 0.611, with RMSE and MAE reduced to 0.258 kg m^−2^ and 0.205 kg m^−2^, respectively, representing improved performance, although still substantially lower than that of traditional machine learning models. The support vector machine (SVM) demonstrated strong predictive capability: the linear kernel SVM attained an R^2^ of 0.760 and an RMSE of 0.203 kg m^−2^, while the radial basis function (RBF) kernel SVM achieved an R^2^ of 0.734 and an RMSE of 0.214 kg m^−2^. Both SVM variants exhibited significantly lower errors compared to neural network models, with the linear kernel SVM outperforming its nonlinear counterpart. The random forest (RF) model produced stable results across different tree counts. With 100 trees, it achieved an R^2^ of 0.769 and an RMSE of 0.199 kg m^−2^; when the number of trees increased to 500, the R^2^ and RMSE were 0.763 and 0.201 kg m^−2^, respectively, suggesting convergence in model performance beyond a certain tree count. The extremely randomized trees (ET) model further enhanced prediction accuracy, achieving an R^2^ of 0.784, with RMSE and MAE decreasing to 0.192 kg m^−2^ and 0.158 kg m^−2^, respectively. Among all the individual models, the HGB model performed best, attaining an R^2^ of 0.798, an RMSE of 0.186 kg m^−2^, and an MAE of 0.152 kg m^−2^, highlighting the effectiveness of boosting algorithms in error reduction.

**Figure 6:**
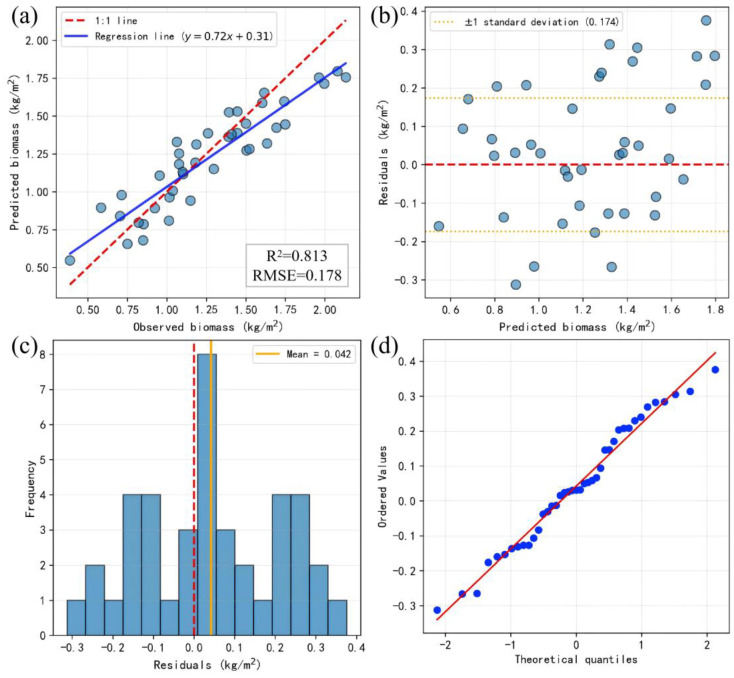
Performance evaluation of the ensemble model for aboveground biomass estimation. (**a**) Observed vs. predicted values; (**b**) residuals vs. predicted values; (**c**) histogram of residuals; (**d**) Q-Q plot of residuals.

The integrated ensemble model (RF + ET + HGB) outperformed all other models, achieving the highest R^2^ of 0.813, with RMSE and MAE further reduced to 0.178 kg m^−2^ and 0.146 kg m^−2^, respectively. These results indicate that ensemble learning approaches surpass individual models in both prediction accuracy and error control, particularly relative to neural network methods, suggesting that ensemble strategies are better suited for high-precision estimation of alfalfa aboveground biomass under the given sample size and feature characteristics. To further assess the predictive accuracy and error structure of the ensemble model on the test set, regression and residual analyses were conducted on its predictions (Figure 6).

The linear regression results between the measured biomass and the predicted value of the model on the test set (Figure 6a) showed that there was a significant linear correlation between the predicted value and the measured value, with the coefficient of determination reaching 0.813 and the root mean square error (RMSE) of 0.178 kg m^−2^, indicating that the overall prediction accuracy of the model was high. The slope of the regression line equation was lower than the ideal 1:1 relationship, indicating that the model had a certain degree of underestimation trend in the high biomass interval, and a slight overestimation in the low biomass interval. Most of the sample points were still closely distributed around the 1:1 line, indicating a good fit ability of the model in the range of the master data. The residuals were randomly distributed around zero values (Figure 6b) and showed no obvious systematic trend or heteroscedasticity. The standard deviation of the residuals was 0.174, and most of the sample residuals were within the range of ±1 standard deviation, indicating that the prediction error was controlled as a whole, and the model maintained a relatively consistent error structure at different biomass levels. This error structure indicates that the model does not exhibit systematic overestimation or underestimation under normal biomass conditions, which enhances its reliability for operational pasture monitoring. In practical applications, such error randomness ensures that prediction deviations are unlikely to accumulate in a specific management direction.

The distribution of residuals in the test set samples was approximately symmetrical (Figure 6c), and the mean value was 0.042, which was close to zero, indicating that there was no significant systematic bias in the model. The skewness and kurtosis of the residual distribution were −0.037 and −0.832, respectively, indicating that the residual distribution was slightly flat but basically consistent with the characteristics of normal distribution. This result further supports the assumption of randomness in the prediction error of the model. Most of the sample points are closely distributed around the theoretical quantile (Figure 6d), and there is only a slight deviation in the tail of the distribution, indicating that the residual overall meets the assumption of normal distribution, and the model prediction results have good statistical stability. In addition, 39.0% of the samples had absolute residuals less than 0.1, and 26.8% of the samples had absolute residuals in the range of 0.1–0.2, with a total of more than 65%. From an agronomic perspective, an absolute error below 0.2 kg·m^−2^ represents a relatively small deviation compared with the overall biomass range observed in this study (mean ≈ 1.26 kg·m^−2^). Therefore, the model accuracy is sufficient to support field-level biomass estimation, harvest timing decisions, and treatment comparison under typical management scenarios. Only 9.8% of the samples had absolute residuals greater than 0.3, and no extreme error values exceeding 0.4 were found, indicating that the model had high prediction reliability on most samples.

### 3.2. Analysis of Model Robustness

**Figure 7:**
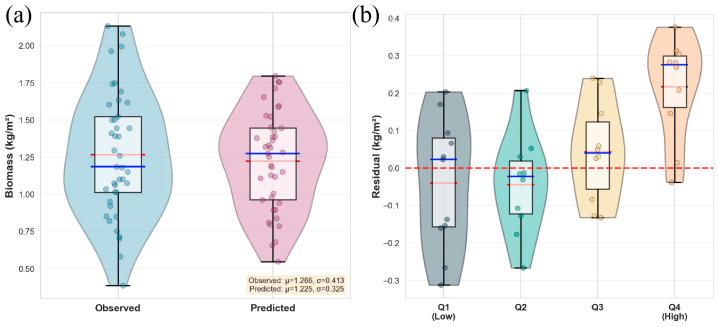
Distribution consistency and residual behavior of the ensemble model predictions. (**a**) Comparison of the distribution of observed and predicted aboveground biomass. The violin plots represent the kernel density distribution, while the embedded boxplots indicate the interquartile range (IQR). The red line represents the median and the blue line represents the mean. (**b**) Distribution of prediction residuals across biomass quartile groups (Q1-Q4). The dashed red horizontal line indicates the zero-residual reference.

The robustness of the model was further tested, the performance of the predicted results at the overall distribution level and under different biomass gradients was analyzed, and the distribution characteristics and grouping residuals of the measured and predicted values were compared and analyzed (Figure 7).

By comparing the distribution characteristics of the measured biomass and the model-predicted biomass (Figure 7a), the overall distribution pattern of the predicted biomass was consistent with the measured value, and the median and quartile intervals of the two were highly overlapping. The mean value of the measured biomass was 1.266, and the standard deviation was 0.413, while the mean value of the predicted biomass was 1.225, and the standard deviation was 0.325, indicating that the predicted biomass was close to the measured value at the mean level, but the dispersion degree was slightly converged. Compared with the measured values, the tail width of the predicted distribution in the extreme high value and low value regions decreased, indicating that the model smoothed the biomass distribution as a whole, reducing the influence of extreme values on the prediction results. This feature was consistent with the mechanism of reducing the variance of the ensemble learning model through multi-model averaging.

The predicted residuals were grouped based on the quartiles of the measured biomass (Q1-Q4) (Figure 7b). The median residuals of the low biomass interval (Q1) and the medium and low biomass interval (Q2) were close to zero, but the distribution range was wide, and the residuals showed positive and negative fluctuations at the same time. The analysis demonstrates that the prediction error of the model in the low value interval had strong randomness. In the middle and high biomass interval (Q3), the residual was still distributed around the zero value, but the distribution shifted slightly to the positive value, indicating that the model began to show a slight underestimation trend in this interval. The distribution of residuals in the high biomass interval (Q4) was significantly skewed to positive values, with the median and mean values higher than zero, and the amplitude of residuals increased, indicating that the underestimation of the model was significantly enhanced under high biomass conditions.

To further clarify which types of prediction errors are reduced by multimodal fusion, biomass samples were stratified into four intervals: low (<1.0 kg/m^2^), medium-low (1.0–1.3 kg/m^2^), medium-high (1.3–1.6 kg/m^2^), and high (>1.6 kg/m^2^). The results reveal an interval-dependent pattern (Table A4). In the medium-high and high biomass ranges, the fusion model achieves the lowest MAE, reducing error by 7.1% and 7.9%, respectively, compared with the multispectral-only model. This indicates that LiDAR-derived structural features help alleviate spectral saturation and systematic underestimation in dense canopy conditions. In contrast, in low biomass intervals, performance differences are small, suggesting that spectral information alone sufficiently captures variability when canopy density is low. Overall, multimodal fusion primarily reduces high-biomass underestimation and improves prediction stability across heterogeneous growth conditions.

**Figure A2:**
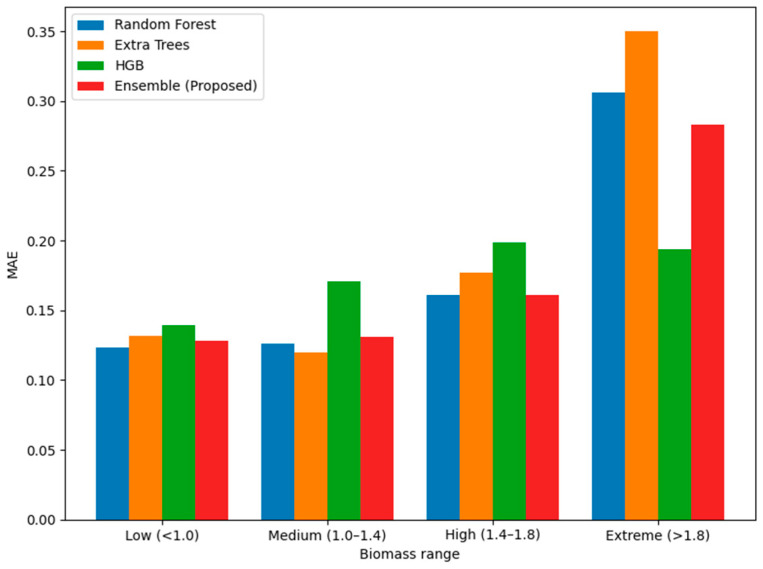
Comparison of prediction errors (MAE) of different models across biomass intervals. All models exhibit increased prediction errors in the high and extreme biomass ranges, indicating that this issue is not model-specific. The proposed ensemble model shows relatively lower errors across all intervals, particularly under extreme biomass conditions.

In conclusion, the model could well reproduce the statistical characteristics of the measured biomass at the overall distribution level, but there were still systematic differences under different biomass gradients. The integrated model had good stability and unbiasedness in the medium and low biomass interval, while its prediction error showed a certain structural shift in the high biomass interval. The comparison of prediction errors across biomass intervals (Figure A2) shows that all models exhibit increased errors under high biomass conditions, indicating that this issue is not model-specific. This phenomenon may be related to the relatively small number of high biomass samples and the limited expression ability of the feature space in the high value interval. According to the distribution consistency analysis and grouped residual analysis, the model had reliable prediction ability in most sample ranges, but there was still room for further optimization in the extreme interval of high biomass.

### 3.3. Effects of Different Index Types on Modeling Accuracy

**Figure 8:**
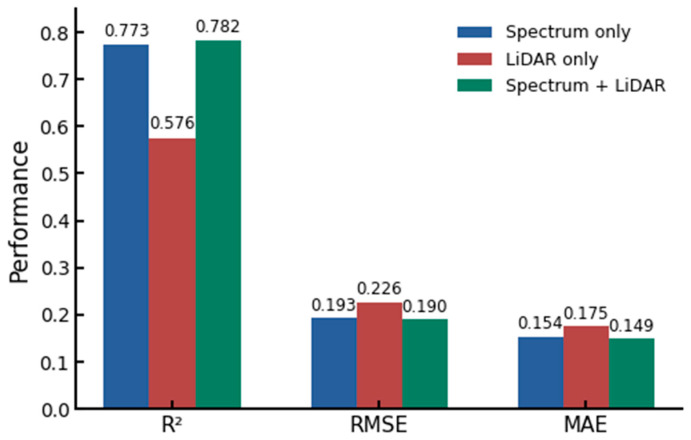
The impact of different indicator types on modeling accuracy.

In order to evaluate the influence of different data sources on the modeling accuracy of biomass, three types of feature combination models were constructed, including only using spectral features, only using LiDAR features, and the integrated model of spectral and LiDAR features (Figure 8). The integrated models were used for modeling respectively.

The results show that the model combined with spectral and LiDAR features (46 features) achieves the highest prediction accuracy, and its optimal R^2^ reaches 0.782, while MAE and RMSE are 0.149 kg m^−2^ and 0.190 kg m^−2^, respectively. The overall performance is better than that of the single data source model. This result indicated that the fusion of multi-source remote sensing data could effectively improve the accuracy of biomass retrieval. The model using only spectral features (42 features) also showed high predictive ability, and the optimal R^2^ was 0.773, which was close to the integrated model, indicating that spectral information played a dominant role in biomass estimation. In contrast, the performance of the model using only LiDAR features (4 features) was significantly lower (R^2^ = 0.576), which may be related to the small number of features and the indirectivity of structural information for biomass representation. However, the canopy structure information provided by LiDAR still has a certain complementary effect on the model performance after being fused with the spectral features.

### 3.4. Comparison of Model Accuracy at Different Growth Times

The prediction performance of the proposed ensemble model under different growth stages was further analyzed. The results indicate that there were significant differences in modeling accuracy among different growth stages (Table 8).

The prediction accuracy of the model showed stage-dependent differences (Table 8), and the model performance exhibited temporal fluctuations. The results for each growth stage are calculated on subsets of the test data. During the early regrowth and branching stages, the model generally performed well, achieving high fitting accuracy with R^2^ values of 0.885 and 0.917, respectively, and relatively low MAE and RMSE. Previous UAV-based biomass studies have reported that spectral features exhibit strong sensitivity to leaf expansion and chlorophyll accumulation during early vegetative stages [42], which likely explains the stable and reliable prediction performance observed in this study.

In contrast, during the bud stage (20 August), the model performance declined significantly (R^2^ = 0.467), while MAE and RMSE increased markedly. The bud stage represents a transitional phase characterized by rapid stem elongation and increasing vertical stratification, which can weaken the direct correspondence between vegetation indices and biomass. Moreover, vegetation-index-based estimation has been shown to experience reduced sensitivity under denser canopy conditions due to partial saturation effects [43], which may have further limited model generalization at this stage.

After the bud stage, prediction accuracy improved during the early flowering stage (R^2^ = 0.802) and remained relatively stable during the full flowering stage (R^2^ = 0.823). Studies incorporating structural or phenological variables have demonstrated improved robustness of biomass estimation across main growth stages [44], suggesting that the structural information used in this study may have contributed to the recovery of prediction performance under more complex canopy conditions.

Overall, the model exhibited higher prediction accuracy during early growth stages and late flowering stage, while prediction performance was relatively weaker during the bud stage. These results indicate that phenology-aware modeling strategies may further enhance biomass prediction stability across heterogeneous growth periods.

### 3.5. Feature Importance Analysis

**Figure 9:**
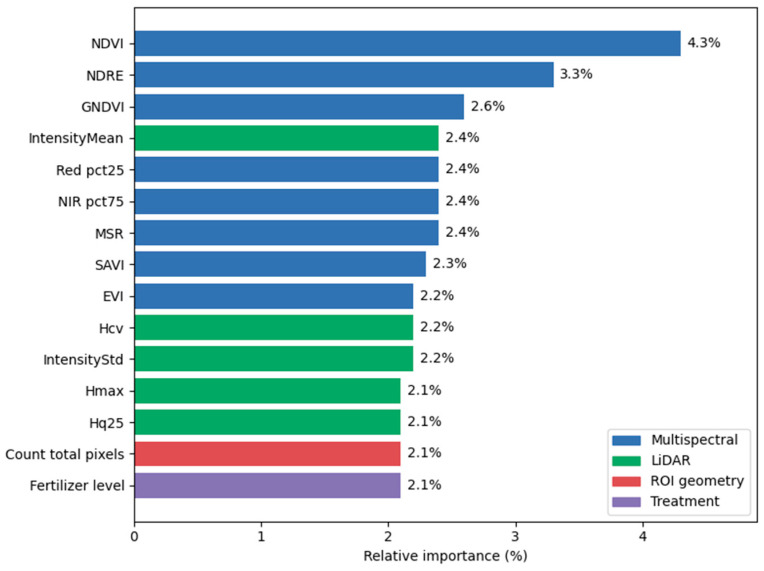
Feature importance ranking. Only the top 15 features (out of 49 total features) ranked by importance are presented.

Under the optimal modeling condition, the relative contribution of each input feature in the model to the prediction result is systematically analyzed to evaluate the importance of different features in the model decision process (Figure 9).

The results show that the model does not rely on a single variable, but achieves high-precision fitting through the synergy of multi-source features. Spectral features (such as NDVI and NDRE) showed the highest contribution rate, accounting for 4.3% and 3.3%, respectively, which verified the central role of physiological activity and pigment content in the estimation of target variables. At the same time, LiDAR structural features (such as Hcv, Hmax) and laser reflection intensity feature IntensityMean also occupied high weights in the biomass prediction process, reaching 2.2%, 2.2%, and 2.4%, respectively. This complementarity of physical structure features and spectral physiological features effectively alleviates the saturation problem of single sensors when the vegetation coverage is high, and improves the prediction accuracy of the model.

**Figure 10:**
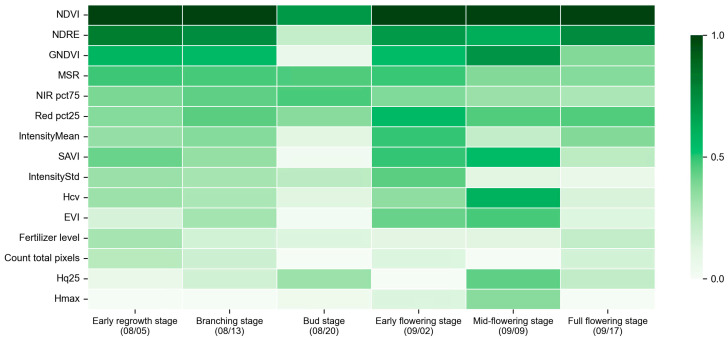
Perform dynamic analysis of normalized feature importance for the model.

To further reveal the dynamic changes in the contribution of different remote sensing and structural features to biomass prediction during the growth process of alfalfa, based on a comprehensive model integrating multispectral and LiDAR data, the dynamic patterns of the relative importance of the top 15 important input features at different growth stages were summarized (Figure 10).

The results showed that the importance of each characteristic exhibited significant dynamic changes across different growth stages. Overall, spectral index features maintained a high contribution throughout the entire growth cycle, with NDVI and NDRE dominating during the early regrowth and branching stages (5 August–13 August), indicating that canopy greenness and chlorophyll content were the primary factors characterizing early biomass accumulation. As growth progressed to the bud stage (20 August), the importance of several multispectral band statistical features increased significantly, reflecting the enhanced ability of spectral distribution characteristics to capture biomass differences under increasing canopy structural complexity. During the early flowering stage and full flowering stage (2 September–17 September), the importance of LiDAR-derived structural features increased markedly, and the contributions of HCV, Hmax to model prediction became more pronounced, indicating that three-dimensional structural information played an important complementary role under medium- and high-biomass conditions. In general, the model was dominated by spectral features during the early growth stages and gradually transitioned to a framework jointly driven by spectral and structural features in the later growth stages, further demonstrating the necessity of multi-source remote sensing data fusion for biomass estimation across different growth stages.

### 3.6. Modeling Comparison of Key Features

**Figure 11:**
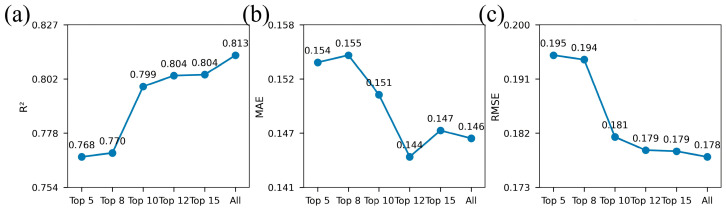
Comparison of different quantitative features and full feature modeling results. Figures (**a**), (**b**) and (**c**) are R^2^, MAE, and RMSE results respectively. The “All” configuration denotes the full feature set (49 features), which corresponds to the ensemble model results reported in Table 7.

In order to systematically discuss the influence of the number of features on the prediction performance of the model, based on the feature importance ranking results, according to the principle from less to more, the Top 5, Top 8, Top 10, Top 12, and Top 15 key features are selected in turn, and compared with the modeling results under the condition of full feature set. The change rules of model accuracy and stability under different feature scales were evaluated (Figure 11).

The number of different features has a significant impact on the prediction performance of the model (Figure 10). On the whole, the performance of the model continues to improve with the increase of the number of features, and the full feature modeling has the best performance in terms of comprehensive prediction ability. When the number of features is small (5–8), the fitting ability of the model is limited, with R^2^ of 0.768 and 0.770, respectively, and MAE and RMSE are at a high level, indicating that it is difficult to fully describe the change characteristics of the target variable by only relying on a small number of features. With the number of features increasing to 12, the performance of the model is significantly improved. The R^2^ increases to 0.799 and 0.804, respectively, and the error index decreases significantly. Among them, the MAE under the Top 12 features reaches the lowest value of 0.144, indicating that the high importance features play a key role in the model prediction. When more features are introduced, the overall performance of the model still shows an upward trend. When using Top 15 features, the R^2^ stabilizes at 0.804 and RMSE decreases to 0.179, which shows that the model’s ability to represent complex information is further enhanced.

Conversely, the full feature model (49 features) performs the best in the comprehensive index, with the highest R^2^ value of 0.813 and the lowest RMSE value of 0.178, indicating that the full feature set can retain the original information to the greatest extent, and effectively improve the overall fitting ability and prediction stability of the model. Although there is little difference between the full feature model and the Top 12-Top 15 feature schemes in MAE (0.146), its overall advantage is more obvious, indicating that the introduction of all features helps the model to capture more subtle but effective information, thereby improving the prediction accuracy. After gradually introducing more features, the overall trend of model performance is gradually improving (Table A2). While the improvement from Top30/Top40 to the full 49-feature set is moderate, the complete feature configuration consistently achieves the best overall performance across all evaluation metrics. This suggests that the additional features provide complementary rather than redundant information, contributing to a more comprehensive representation of alfalfa canopy characteristics. Therefore, the full 49-feature set was retained to achieve a better balance between model complexity and predictive generalization. In general, the full feature model has obvious advantages in prediction performance and stability, while the feature screening model has certain application value in reducing the dimension and improving efficiency.

## 4. Discussion

To evaluate the computational efficiency of the proposed multimodal framework, runtime analysis was conducted on a workstation running Ubuntu 20.04, equipped with an AMD Ryzen 9700X processor, 64 GB RAM (Table A3). The average end-to-end processing time per sample was approximately 6.3 ± 0.8 s, based on 50 samples. Among these steps, multispectral vegetation index computation required 1.8 ± 0.3 s, LiDAR point cloud structural feature extraction required 4.5 ± 0.7 s, while model inference required only 23 ± 5 milliseconds. The primary computational cost lies in point cloud voxelization and convex hull computation. Batch processing of 328 samples required approximately 35 min. These results indicate that the model inference stage is lightweight, and the computational burden is mainly associated with feature extraction. With further optimization such as GPU acceleration or lightweight implementation, the proposed framework has strong potential for near-real-time operational deployment.

The applicability of the proposed framework under different planting densities and alfalfa varieties deserves further discussion. Although the initial sowing rate was consistent across plots, the experimental design included three distinct alfalfa varieties and multiple fertilization gradients, resulting in substantial variation in canopy density, structural heterogeneity, and biomass accumulation patterns. The integration of spectral and LiDAR-derived structural features enables the model to capture both physiological and morphological differences among varieties and density conditions. In particular, the stratified residual analysis indicates that multimodal fusion improves prediction stability in high-density canopy scenarios. However, this study was conducted within a single ecological region. Future work will incorporate explicitly controlled density treatments and multi-regional trials to comprehensively assess cross-density and cross-variety transferability.

In this study, the ensemble learning model based on UAV multispectral imagery and airborne LiDAR data achieved high accuracy (R^2^ = 0.813) in the prediction of alfalfa aboveground biomass. Compared with existing studies, this result is at a high level in the field of remote sensing inversion of forage and crop biomass, and shows strong stability and generalization ability under complex field environments and limited sample conditions.

Using machine learning to predict crop biomass has always been the focus of research. Lucero et al. [39] constructed an alfalfa biomass prediction model based on satellite multispectral data, and the coefficient of determination of the prediction was about 0.61, which was mainly limited by the lack of spatial resolution and canopy structure information. In contrast, the LiDAR data used in this study improved the problem of insufficient spatial resolution and compensated for the limitations from the perspective of data sources. Yang et al. [40] used multispectral data and nonlinear model to invert alfalfa leaf area index. Although good results were obtained in the local time phase, the overall R^2^ was mostly concentrated in the range of 0.60–0.70, which made it difficult to reflect the biomass change stably. In contrast, in this study, the introduction of 3D structural features of LiDAR effectively made up for the lack of easy saturation of single spectral data under high biomass conditions, thereby significantly improving the prediction accuracy. Güner et al. [42] used the artificial neural network (ANN) to predict crop biomass, and its R^2^ was between about 0.50–0.65, which was highly sensitive to sample size. In this study, an ensemble learning model was constructed to give full play to the advantages of each model and significantly improve the prediction accuracy. Abdolrasol et al. [43] found that ANN and DNN models often suffer from unstable convergence and over-fitting problems when the number of samples is limited or the feature dimension is high. In the comparative experiment of this study, the R-squared of ANN and DNN models were only 0.413 and 0.611, respectively, which further verified the above conclusions. Although Support Vector Machine (SVM) performs well in some studies, its performance is highly sensitive to the choice of kernel function and parameter setting. El Kafrawy et al. [44] show that SVM is easily affected by noise in high-dimensional feature space, which leads to a decrease in generalization ability. In this study, the R-squared of the SVM model was 0.734–0.760, which was still significantly lower than that of the ensemble model.

Compared with the above studies, the advantages of this study are mainly reflected in three aspects. Firstly, by fusing multispectral and LiDAR data, the canopy characteristics of alfalfa were systematically characterized from two complementary dimensions: spectral response and three-dimensional structure, which effectively alleviated the problems of spectral saturation and structural information loss. Secondly, a heterogeneous integrated framework composed of random forest, extra trees, and the HGB model was used to make full use of the complementarity of different algorithms in bias-variance control, which improved the stability and generalization ability of the model. Thirdly, under the condition of relatively limited sample size, the ensemble model still maintains high prediction accuracy and shows good adaptability to small sample size.

Although the overall performance of the model in this study is excellent, there is still a certain degree of underestimation in the high biomass interval, which is consistent with the conclusion that the prediction uncertainty increases under the condition of high canopy density in previous studies [28]. Future studies can further improve the prediction ability of the model under extreme growth conditions by increasing the proportion of high biomass samples, introducing multiple time series features, or combining physical constraint models.

The proposed framework demonstrates high prediction accuracy, but its practical application may be constrained by the requirement for UAV platforms, LiDAR sensors, and data processing expertise. Therefore, it may not be directly applicable for small-scale farmers. In practice, this approach is more suitable for agricultural service providers, research institutions, and breeding programs, where remote sensing equipment and technical infrastructure are available. For example, the proposed method can be applied in large-scale pasture monitoring, precision management of forage production, and variety evaluation in breeding trials, where accurate biomass estimation is essential for decision-making. In addition, government agencies and agricultural enterprises can utilize such technologies for regional monitoring and yield assessment.

It is worth noting that the model inference stage is computationally efficient, and the main computational cost lies in feature extraction. Therefore, future work will focus on simplifying the data acquisition process, such as using multispectral imagery alone or low-cost UAV platforms, and developing lightweight deployment frameworks. These improvements will help promote the practical application and commercialization of biomass prediction models in real-world agricultural scenarios.

## 5. Conclusions

In this study, an ensemble learning approach integrating UAV multispectral imagery and airborne LiDAR data was developed to estimate alfalfa aboveground biomass. A multimodal feature system was constructed and multiple machine learning models (random forest, extra trees, and HGB) were combined to capture the nonlinear relationships between features and biomass. The integrated model achieved a coefficient of determination (R^2^) of 0.813 on the test set, with RMSE and MAE values of 0.178 kg m^−2^ and 0.146 kg m^−2^, respectively. Compared with individual models, the ensemble approach provided consistent improvements in predictive accuracy. Error analysis further showed that the model produced reasonable residual distributions without obvious systematic bias. The prediction performance was stable in low and medium biomass ranges, although a slight underestimation was observed under high biomass conditions. These results indicate that integrating multisource UAV data with ensemble learning can provide an effective approach for non-destructive estimation of alfalfa biomass. Future work may further improve model performance by increasing the representation of high biomass samples and incorporating multi-temporal information.

## Figures and Tables

**Table 1:** Specific parameters of unmanned aerial vehicles.

System	Type	Resolution	Characteristics	Output Data	Accuracy
Zenmuse L2 (LiDAR)	Active laser scanning	Point cloud rate: 1.2 M pts/s (multi-return)	905 nm laser	3D point cloud (XYZI), depth map	5 cm (planar), 4 cm (vertical) @150 m
RedEdge-P (multispectral)	Passive multispectral imaging	1.6 MP × 5 bands; 5.1 MP panchromatic; GSD 2 cm @60 m	Blue 475 nm, Green 560 nm, Red 668 nm, Red-edge 717 nm, NIR 842 nm	Reflectance imagery (5 bands), pan-sharpened image	Geometric accuracy depends on RTK

**Table 2:** Data splicing and composition results.

Type	Numbers	Format	Size	Description	Application
multispectral Imagery	15	TIF	32.8 GB	Five spectral bands: Blue, Green, Red, Red Edge, and NIR	Spectral reflectance analysis and vegetation index computation
LiDAR Point Cloud	15	LAS	422.5 GB	Point density of 150–200 pts/m^2^; includes intensity and additional attributes	Extraction of point-density and elevation-based features

**Table 3:** Description of ROI partitioning data.

Plot	Number of Data Batches	ROIs per Batch	Total ROIs	Valid Samples
CY3H	6	54	324	108
XM4H	4	54	216	72
ZM10H	5	54	270	90
Total	15	162	810	270

**Table 4:** Calculation formula of some important indices.

Vegetation Indices	Calculation Formula
NDVI	$\mathrm{NDVI}=\frac{NIR-Red}{NIR+Red}$
NDRE	$\mathrm{NDRE}=\frac{NIR-RedEdge}{NIR+RedEdge}$
GNDVI	$\mathrm{GNDVI}=\frac{NIR-Green}{NIR+Green}$
MSR	$\mathrm{MSR}=\frac{\left(\frac{NIR}{Red}-1\right)}{\sqrt{\left(\frac{NIR}{Red}+1\right)}}$
EVI	$\mathrm{EVI}=2.5\cdot \frac{NIR-Red}{NIR+6\cdot Red-7.5\cdot Blue+1}$
SAVI	$\mathrm{SAVI}=1.5\cdot \frac{NIR-Red}{NIR+Red+L}$

**Table 5:** Modeling features.

Feature Type	Representative Features	Quantity	Description
multispectral features	NDVI, NDRE, GNDVI, MSR, EVI, SAVI	42	Pixel statistics, quantiles, and distribution characteristics from 5 bands (Blue/Green/Red/RedEdge/NIR).
LiDAR features	IntensityMean, LiDAR_area_m2, IntensityStd, Hcv	4	Based on the normalization of point cloud convex hull area and height, it mainly reflects the variation of echo intensity, structural area, and canopy morphology.
ROI geometric features	roi_area_m2, count_total_pixels	2	Characterize the area and coverage of the sample plot to express data integrity and scale information.
Handling variables	Fertilizer level	1	Independent variables related to experimental treatment, used to characterize the effect of treatment level on biomass.

**Table 6:** Model optimization parameter.

Model	Hyperparameter	Value	Description
Random Forest	n_estimators	1000	Number of trees
	max_depth	15	Maximum tree depth
	min_samples_split	5	Minimum samples required to split an internal node
	min_samples_leaf	2	Minimum samples required at a leaf node
	max_features	sqrt	Maximum number of features considered at each split
Extra Trees	n_estimators	1200	Number of trees
	max_depth	None	Unlimited tree depth
	min_samples_split	4	Minimum samples required to split an internal node
	min_samples_leaf	1	Minimum samples required at a leaf node
	max_features	sqrt	Maximum number of features considered at each split
	bootstrap	False	Whether bootstrap sampling is used
HGB	max_iter	1000	Maximum number of iterations
	learning_rate	0.05	Learning rate
	max_depth	8	Maximum tree depth
	min_samples_leaf	5	Minimum samples required at a leaf node
	l2_regularization	1	L2 regularization parameter

**Table 7:** Comparison of prediction performance of different models.

Model	R^2^	RMSE	MAE
ANN	0.413	0.318	0.234
DNN	0.611	0.258	0.205
SVM-Linear	0.760	0.203	0.170
SVM-RBF	0.734	0.214	0.160
Random Forest (500 trees)	0.763	0.201	0.150
Random Forest (100 trees)	0.769	0.199	0.164
Extra Trees	0.784	0.192	0.158
HGB	0.798	0.186	0.152
Ensemble (RF + ET + HGB)	0.813	0.178	0.146

**Table 8:** Prediction accuracy of the model under different growth stages.

Stages	R^2^	MAE	RMSE
Early regrowth stage	0.885	0.070 kg m^−2^	0.086 kg m^−2^
Branching stage	0.917	0.034 kg m^−2^	0.040 kg m^−2^
Bud stage	0.467	0.142 kg m^−2^	0.183 kg m^−2^
Early flowering stage	0.802	0.043 kg m^−2^	0.049 kg m^−2^
Mid-flowering stage	0.621	0.117 kg m^−2^	0.122 kg m^−2^
Full flowering stage	0.823	0.074 kg m^−2^	0.091 kg m^−2^

## Data Availability

All data and materials are available upon request to QL (qingli@njau.edu.cn).

## Appendix A

**Table A1:** Model performance under repeated random train-test splits (85–15%) using different random seeds.

Split	R^2^	RMSE	MAE
Seed 1	0.813	0.178	0.146
Seed 2	0.805	0.182	0.151
Seed 3	0.821	0.174	0.143
Seed 4	0.809	0.181	0.149
Seed 5	0.816	0.176	0.147

**Table A2:** Performance comparison under different feature subsets.

Features	R^2^	RMSE	MAE
Top10	0.799	0.181	0.151
Top20	0.806	0.179	0.148
Top30	0.804	0.180	0.149
Top40	0.809	0.179	0.147
Full	0.813	0.178	0.146

**Table A3:** Computational cost analysis of the proposed multimodal data fusion framework.

Processing Stage	Mean Time (s)	Std (s)	Percentage of Total (%)	Remarks
Multispectral vegetation index computation	1.8	0.3	28.6%	Includes reflectance preprocessing and VI calculation
LiDAR structural feature extraction	4.5	0.7	71.4%	Voxelization and convex hull computation are main bottlenecks
Model inference (ensemble prediction)	0.023	0.005	<1%	Tree-based regression prediction
Total per sample	6.3	0.8	100%	End-to-end processing time

**Table A4:** Stratified error comparison among multispectral-only, LiDAR-only, and fusion models across biomass intervals. Biomass samples were stratified into four intervals to examine interval-specific error characteristics. Fusion Improvement (%) is calculated relative to the multispectral-only model. Positive values indicate error reduction achieved by multimodal fusion.

Biomass Interval (kg/m^2^)	Multispectral MAE	LiDAR MAE	Fusion MAE	Fusion Improvement (%)
Low (<1.0)	0.150	0.191	0.147	+2.0%
Medium-low (1.0–1.3)	0.135	0.162	0.143	−5.9%
Medium-high (1.3–1.6)	0.141	0.225	0.131	+7.1%
High (>1.6)	0.241	0.309	0.222	+7.9%

**Table A5:** Complete list of features used for model development.

Feature Name	Description	References
NDVI	$\mathrm{NDVI}=\frac{NIR-Red}{NIR+Red}$	[45]
NDRE	$\mathrm{NDRE}=\frac{NIR-RedEdge}{NIR+RedEdge}$	[45]
Hmax	Maximum crop height (from LiDAR or DSM)	[45]
Hmean	Mean crop height	[45]
Hstd	Height standard deviation	[45]
Hcv	Height coefficient of variation	[45]
EVI	$\mathrm{EVI}=2.5\cdot \frac{NIR-Red}{NIR+6\cdot Red-7.5\cdot Blue+1}$	[45]
RedEdge_std	Standard deviation of Red Edge reflectance	[45]
roi_area_m2	Region of interest area size (image)	[45]
GNDVI	$\mathrm{GNDVI}=\frac{NIR-Green}{NIR+Green}$	[46]
MSR	$\mathrm{MSR}=\frac{\left(\frac{NIR}{Red}-1\right)}{\sqrt{\left(\frac{NIR}{Red}+1\right)}}$	[46]
SAVI	$\mathrm{SAVI}=1.5\cdot \frac{NIR-Red}{NIR+Red+L}$	[46]
Blue_mean	Mean reflectance in Blue band	[46]
Green_mean	Mean reflectance in Green band	[46]
Red_mean	Mean reflectance in Red band	[46]
RedEdge_mean	Mean reflectance in Red Edge band	[46]
NIR_mean	Mean reflectance in Near-Infrared band	[46]
Blue_std	Standard deviation of Blue reflectance	[46]
Green_std	Standard deviation of Green reflectance	[46]
Red_std	Standard deviation of Red reflectance	[46]
Red_pct10	10th percentile reflectance in Red band	[46]
Red_pct25	25th percentile reflectance in Red band	[46]
Red_pct90	90th percentile reflectance in Red band	[46]
NIR_std	Standard deviation of NIR reflectance	[46]
count_total_pixels	Total pixel count in ROI	[46]
Fertilizer_level	Fertilizer application treatment level	[46]
RedEdge_median	Median reflectance in Red Edge band	[47]
density_veg_pt_m2	Vegetation point density per m^2^	[47]
IntensityMean	Mean LiDAR intensity	[47]
IntensityStd	Std of LiDAR intensity	[47]
LiDAR_area_m2	LiDAR derived area	[47]
Hq25	25th percentile crop height	[47]
Hq50	50th percentile crop height	[47]
Hq90	90th percentile crop height	[47]
RedEdge_pct10	10th percentile reflectance in Red Edge	[47]
RedEdge_pct25	25th percentile reflectance in Red Edge	[47]
RedEdge_pct90	90th percentile reflectance in Red Edge	[47]
NIR_pct25	25th percentile reflectance in NIR band	[48]
NIR_pct75	75th percentile reflectance in NIR band	[48]
NIR_pct90	90th percentile reflectance in NIR band	[48]
Green_pct90	90th percentile reflectance in Green band	[48]
NIR_median	Median reflectance in NIR band	[48]
NIR_pct10	10th percentile reflectance in NIR band	[48]
Hiqr	Interquartile range of crop height	[48]
Blue_median	Median reflectance in Blue band	[48]
Green_median	Median reflectance in Green band	[48]
Red_median	Median reflectance in Red band	[48]
coverage_pixels	Vegetation pixel count in ROI	[48]
cover_above_0_3m	Crop cover above 0.3 m	[48]
